# Supplementary material for: Effective Method for the Determination of the Unit Cell Parameters of New MXenes
Source: Materials (Basel). 2022 Dec 9;15(24):8798. doi: 10.3390/ma15248798 (PMC9783200; doi:10.3390/ma15248798)
Supplement: Supplementary file 1 [file materials-15-08798-s001.zip › materials-2060401-supplementary.pdf]

Supplementary material for:

Effective Method for the Determination of the Unit Cell Parameters of New MXenes

Alexander Syuy [1,2,*], Dmitry Shtarev [1,*], Alexey Lembikov [1], Mikhail Gurin [1], Ruslan Kevorkyants [3], Gleb Tcelikov [2], Aleksey Arsenin [2] and Valentyn Volkov [2]

[1] Institute of High Technologies and Advanced Materials, Far Eastern Federal University, 690922 Vladivostok, Russia
[2] Center for Photonics and 2D Materials, Moscow Institute of Physics and Technology, 141701 Dolgoprudny, Russia
[3] Hong Kong Quantum AI Lab Ltd., Hong Kong Science and Technology Parks Corporation, Hong Kong, China
* Correspondence: alsyuy271@gmail.com (A.S.); shtarev@mail.ru (D.S.)

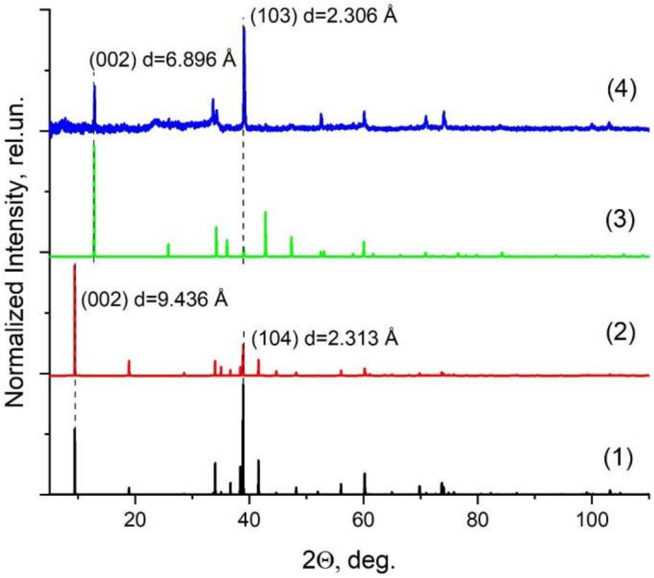

Figure S1. Diffractograms of (1) parent MAX-phase Ti3AlC2, (2) MXene Ti3C2 in the parent MAX-phase Ti3AlC2 geometry, (3) MXene Ti3C2 shrunk along c-axis, and (4) synthesized MXene.

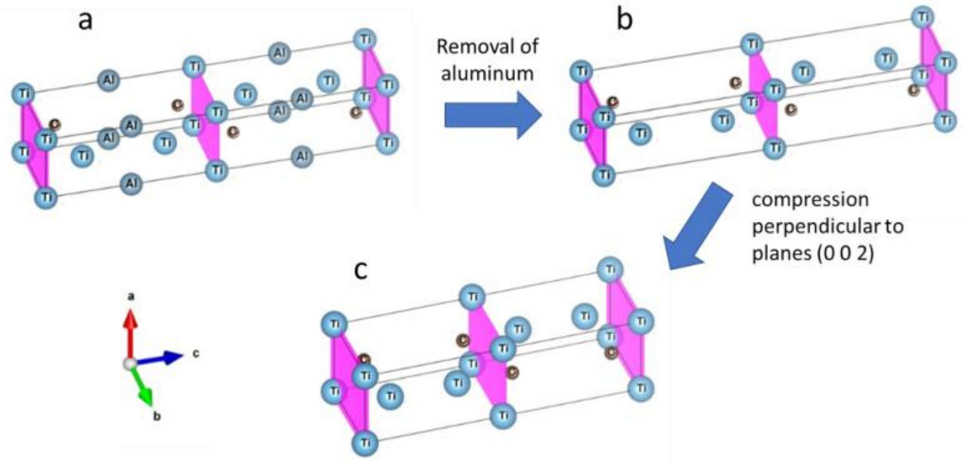

Figure S2. The process of crystal lattice formation of the MXene synthesized from the parent MAX-phase Ti3AlC2: (a) parent MAX-phase Ti3AlC2, (b) parent MAX-phase Ti3AlC2 without Al toms (Ti3C2), and (c) final Ti3C2 structure compressed along the pink (002) planes.

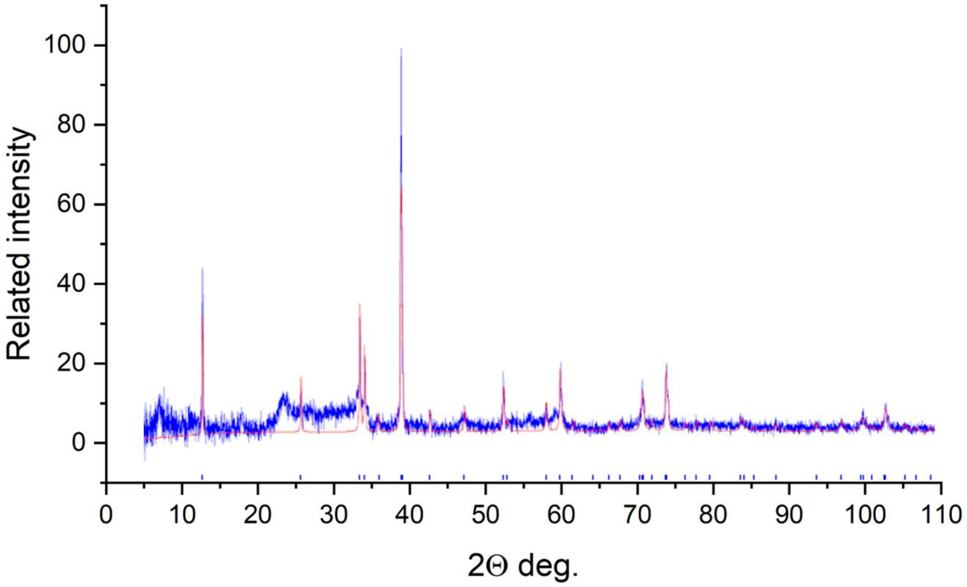

Figure S3. Le Bail fitting of the experimental diffraction data.

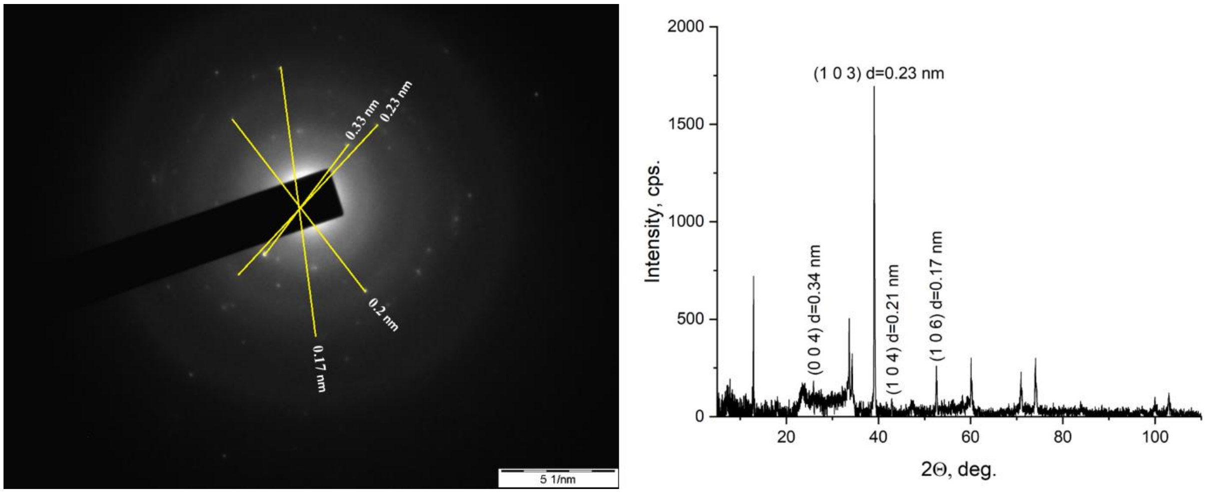

Figure S4. Interplane distances in the synthesized TiNbC MXene. Left: transmitted electrons (TEM). Right: reflected electrons (XRF).

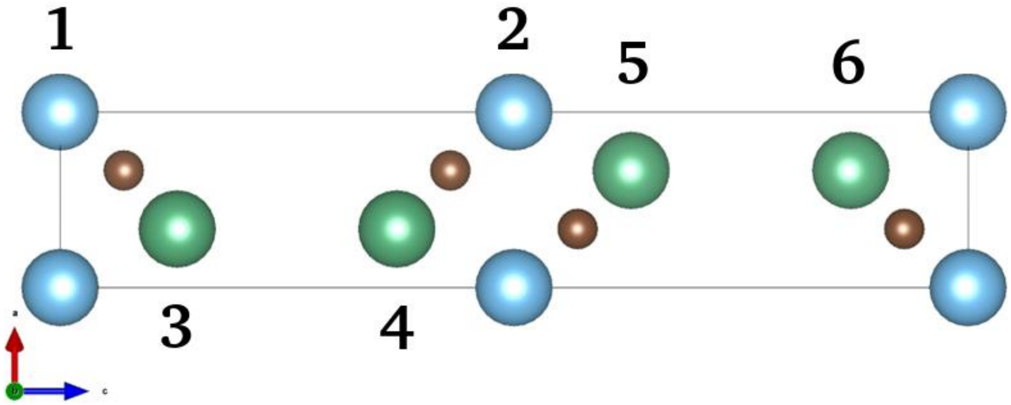

Figure S5. Atomic position numbering in the cationic sublattice of the studied MXene $Ti_2Nb_4C_4$.

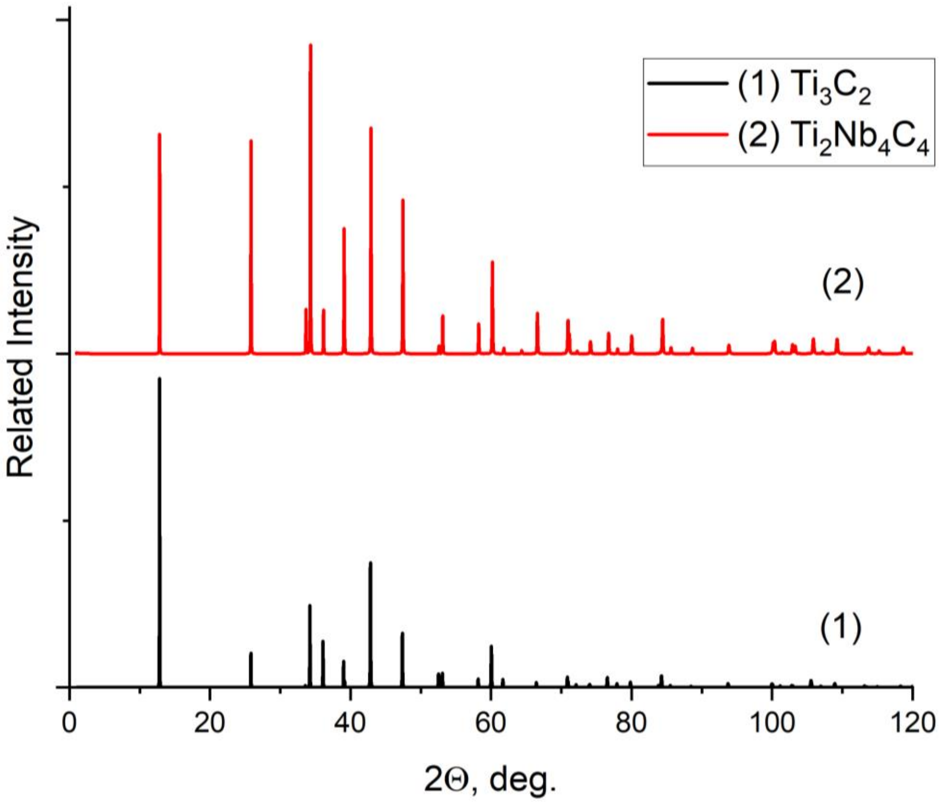

Figure S6. Comparison of calculated XRD patterns before (1) and after (2) substitution of a part of titanium atoms with niobium.
